# On some morphological and ultrastructural features of the insemination system in five species of the genus *Brevipalpus* (Acari: Tenuipalpidae)

**DOI:** 10.1007/s10493-020-00526-x

**Published:** 2020-08-03

**Authors:** Antonella Di Palma, Aline D. Tassi, Elliot W. Kitajima

**Affiliations:** 1 Department of the Sciences of Agriculture, Food and Environment, University of Foggia, 71100 Foggia, Italy; 2 Departamento de Fitopatologia e Nematologia, Escola Superior de Agricultura Luiz de Queiroz (ESALQ), Universidade de São Paulo (USP), Piracicaba, SP 13418-900 Brazil

**Keywords:** Spermatheca, Taxonomy, Identification, Electron microscopy

## Abstract

The genus *Brevipalpus* (Tenuipalpidae) includes 291 described species commonly found in the tropical and subtropical regions. Morphological characters considered in the taxonomy of *Brevipalpus* species are difficult to discern, which often leads to erroneous identifications and the presence of cryptic species within species is suspected. New morphological characters are now considered relevant for identification of *Brevipalpus* species; among them, the morphology of the seminal receptacle (spermatheca) of the female insemination system. This feature has not been considered relevant until now; thus, there is little information about the insemination system in the available species descriptions. Hence, in the present study, ultrastructural details are provided for the insemination system in five species of *Brevipalpus*, representing different morphological groups. The seminal receptacle (spermatheca) and the insemination duct are illustrated using light, transmission and scanning electron microscopy. The spermatheca proved to have specific morphological features that can be useful for taxonomic purposes. On the other hand, its appearance within a population might be variable in a way that needs to be ascertained and evaluated.

## Introduction

The genus *Brevipalpus* belongs to the family Tenuipalpidae (Acari: Prostigmata) and includes 291 described species (Mesa et al. 2009; Castro et al. 2019) commonly found in the tropical and subtropical regions. This genus has great economic importance within the family, because some species were found to transmit phytopathogenic viruses such as *Citrus leprosis virus C* (CiLV-C), *Coffee ringspot virus* (CoRSV), *Passion fruit green spot virus* (PfGSV) and *Orchid fleck virus* (OFV) (Kitajima et al. 2014). In particular, three species have been regarded as plant virus vectors: *Brevipalpus californicus* (Banks), *Brevipalpus obovatus* Donnadieu and *Brevipalpus phoenicis* (Geijskes) (Childers et al. 2003).

Regarding species identification, several morphological characters (e.g., reticulation and details on the cuticle patterns in specific regions of the mite body) used in the taxonomy of *Brevipalpus* are rather difficult to discern, which often leads to erroneous or uncertain identifications. In addition, the appearance of some of these characters may be affected by the age of individuals, diet and mounting techniques (Welbourn et al. 2003). Recent papers reviewing the genus *Brevipalpus*, based on both morphological features (Beard et al. 2015) and molecular markers of the mitochondrial cytochrome oxidase I (COI) gene (Rodrigues et al. 2004; Groot and Breeuwer 2006; Navia et al. 2013), suggested a greater diversity of species, showing some incongruities in the morphological descriptions, when compared with molecular markers, and the possible presence of cryptic species. Therefore, a revaluation of the status of *Brevipalpus* species has become necessary.

In particular, new morphological characteristics are now considered relevant for the identification of *Brevipalpus* species; among them, the morphology of the seminal receptacle (spermatheca) of the female insemination system (Ochoa et al. 2011; Beard et al. 2015). The shape of the spermatheca has already been used for discerning mite species in Phytoseiidae (e.g., Chant and McMurtry 2007). Moreover, attempts have been made to use the morphology of this structure to separate species in some plant mites (e.g., Vacante 1983, 1984; Beard et al. 2015) and this structure morphology could also be correlated to molecular phylogeny lineages suggesting it is evolutionarily conserved in groups like Eriophyoidea (Chetverikov et al. 2015, 2020; Chetverikov and Petanović 2016).

Nevertheless, this character has not been used in the identification of *Brevipalpus* species. In a rather old paper (Castagnoli 1974) there is the first reference to the presence of a spermatheca in the Tenuipalpidae, including the description of this structure in eight species. Later, Baker and Tuttle (1987) described the shape of the spermatheca in some *Brevipalpus* species from Mexico. Yet, for most of the described *Brevipalpus* species, the authors did not observe this structure, sometimes ascribing this to the clearing action of the mounting medium and the age of the females (Baker and Tuttle 1987). In any case taxonomic papers with species descriptions of Tenuipalpidae do not refer to the spermatheca. In Ochoa et al. 2011 suggested the possibility to use this structure in the taxonomy of *Brevipalpus*, and Beard et al. (2015) used the morphology of the spermatheca as a characteristic helping in separation of some species from the *B. phoenicis* complex. In fact, *B. phoenicis sensu lato* was divided into at least eight species (Beard et al. 2015) on the basis of morphological data, using shape of the spermatheca as one of the key characters. Validation of these characters by DNA analysis is still in progress.

Hence, in the present study morphological and ultrastructural details of the insemination system (including the spermatheca) are compared in females of five *Brevipalpus* species. These species represent different morphological groups and are studied using light (LM), transmission (TEM) and scanning electron microscopy (SEM). The aim is to verify, by means of SEM high magnification images, whether the morphological differences observed by LM are confirmed and whether variations in the shape of the spermatheca can be a species-specific character usable in discerning *Brevipalpus* species.

## Materials and methods

Females of the following species were investigated: *Brevipalpus papayensis* (Baker), *B. obovatus*, *Brevipalpus yothersi* (Baker), *B. californicus* and *Brevipalpus tuberellus* De Leon. All these species are considered reproduced parthenogenetically and, according to Baker and Tuttle (1987), *B. yothersi* and *B. papayensis* are representative of the morphological species group *B. phoenicis*; *B.californicus* of the *B. californicus* group; *B. tuberellus* of the *B. cuneatus* group; and *B. obovatus* of the *B. obovatus* group. These five species represent four morphological groups corresponding to molecular clades (Navia et al. 2013; Alves et al. 2019).

*Brevipalpus papayensis* was obtained from a single female collected from a coffee orchard in the State of São Paulo, Brazil (22° 52′ 00.7″ S, 47° 04′ 53.2″ W), starting date 08/05/2018. *Brevipalpus obovatus* population was obtained from a single female collected from *Solanum violifolium* Schott in São Paulo state (22° 42′ 46.9″ S, 47° 38′ 52.4″ W), starting date 03/02/2018. *Brevipalpus yothersi* population was obtained from a single female collected from *Citrus sinensis* L. in São Paulo state (22° 42′ 33.4″ S, 47° 38′ 05.5″ W), starting date 15/08/2016.

**Fig. 1:**
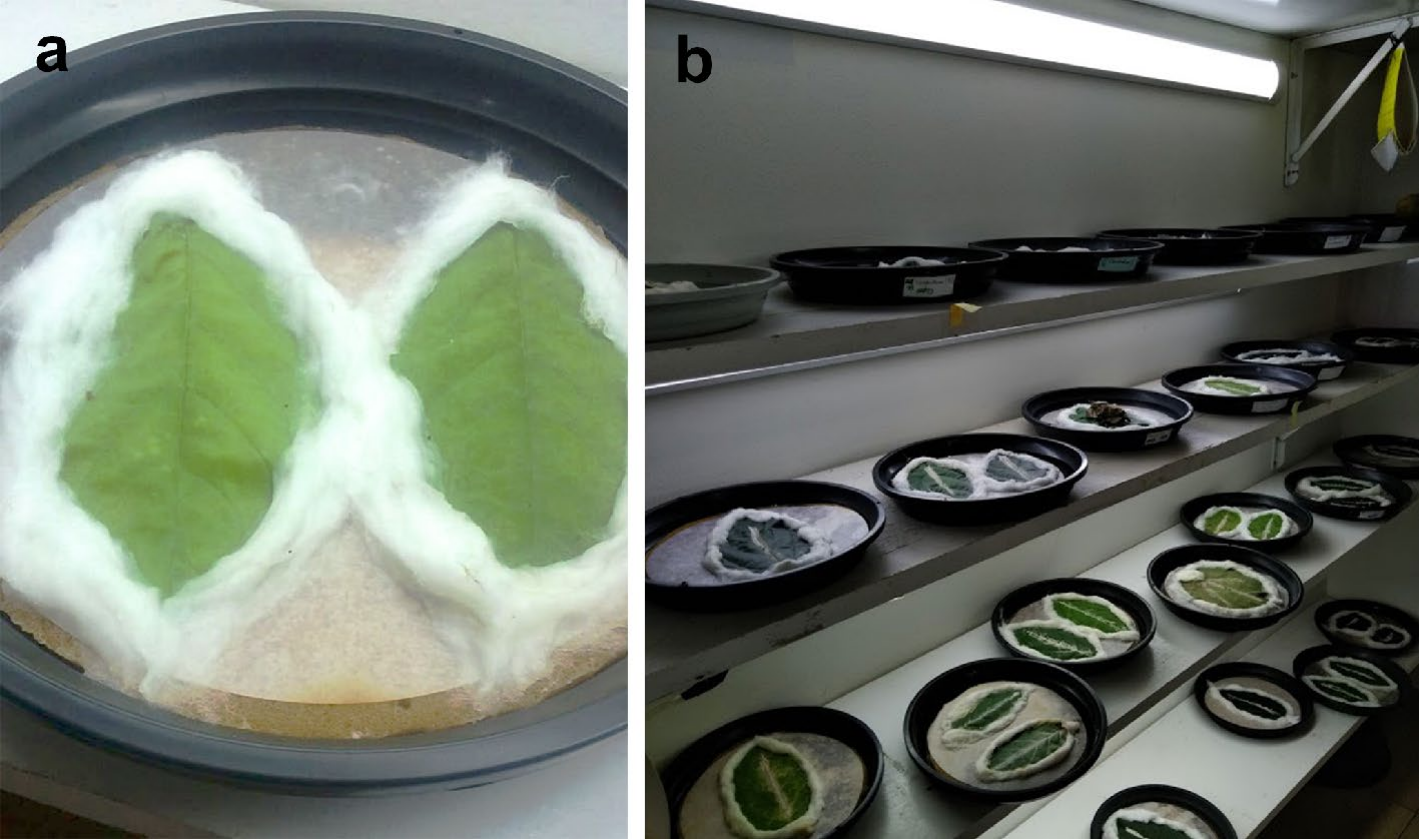
Rearings of *Brevipalpus* species. **a** Picked leaves used as rearing arenas. Their margins are delimited by wet cotton to prevent mites from running away. Leaves lie on the top of a water-soaked sponge covered with filter paper. **b** Rearings in a climatized room

Rearings of these three species were performed at the ESALQ (Luiz de Queiroz College of Agriculture) Piracicaba (São Paolo, Brazil) and realized on picked off leaves of the specific host plants (coffee, citrus and *S. violifolium*) following a modification of the method described by Overmeer (1985). The leaves were used as rearing arenas with their margins delimited by wet cotton to prevent mites from running away. Leaves were put on top of a water-soaked sponge covered with filter paper (Fig. 1a). This procedure allowed the leaves to last for about 1 month. The rearings were kept in a climatized room at 26 ± 2 °C, 65 ± 10% relative humidity and 14:10 h L:D photoperiod (Fig. 1b).

Females of *B. californicus* were collected from *Oncidium* sp. in São Paulo state (22° 42′ 33.7″ S, 47° 38′ 08.7″ W), date 13/09/2018 and stored in 100% ethanol until their use. Females of *B. tuberellus* were collected from *Nectandra megapotamica* (Spreng.), in São Paulo state (22° 42′ 33.3″ S, 47° 38′ 07.3 W), date 25/09/2019, and processed for this study.

### Light microscopy (LM)

Specimens (n = 25) of all listed species were mounted in Hoyer’s medium (Walter and Krantz 2009) for LM. Observations and micrographs were performed with a Zeiss Axioimager II light microscope provided with DIC and Zeiss digital camera.

### Transmission electron microscopy (TEM)

Specimens of *B. papayensis*, *B. yothersi* and *B. tuberellus* (n = 25) were processed for TEM. Females of these species were dissected and prefixed in ice-cold Karnovsky’s (1965) for 3 h. After prefixation, specimens were rinsed in buffer solution (cacodylate buffer pH 7.3) for 2 h, subsequently post-fixed in 2% buffered OsO4 (1.5 h), rinsed again in buffer solution (2 h), dehydrated with graded ethanol up to 100% ethanol, embedded in Spurr’s mixture (Spurr 1969) and finally polymerized for 48 h at 65 °C. Ultrathin serial sections were cut with a Leica EM UC6 ultramicrotome using a diamond knife (Diatome). The sections, collected with copper grids covered with a formvar film, were double-stained with uranyl acetate and lead citrate according to Reynolds et al. (1963) and studied with a JEOL JEM-1011 TEM, images being recorded digitally.

### Scanning electron microscopy (SEM)

To obtain three-dimensional pictures in high magnification of the insemination system, a methodology already applied for the same purpose to other groups of mites was used (Di Palma and Alberti 2001; Alberti et al. 2009).

Some specimens (n = 45) of all listed species were immersed in lactic acid for around 2 weeks at 50 °C to dissolve the soft tissues present in the mite body while preserving the cuticle-lined structures. When the mites were completely clarified (translucent) they were dehydrated in graded ethanol and critical point dried in a Leica EM CPD300 critical point dryer. Then the specimens were placed on Al stubs using double sticking carbonated tapes and, using a fine needle, the dorsal shields were removed to expose the insemination system. Then the specimens were sputter-coated with gold in a Baltec SCD050 sputter coater and studied with a JEOL IT300 SEM. Images were registered digitally.

## Results

**Fig. 2:**
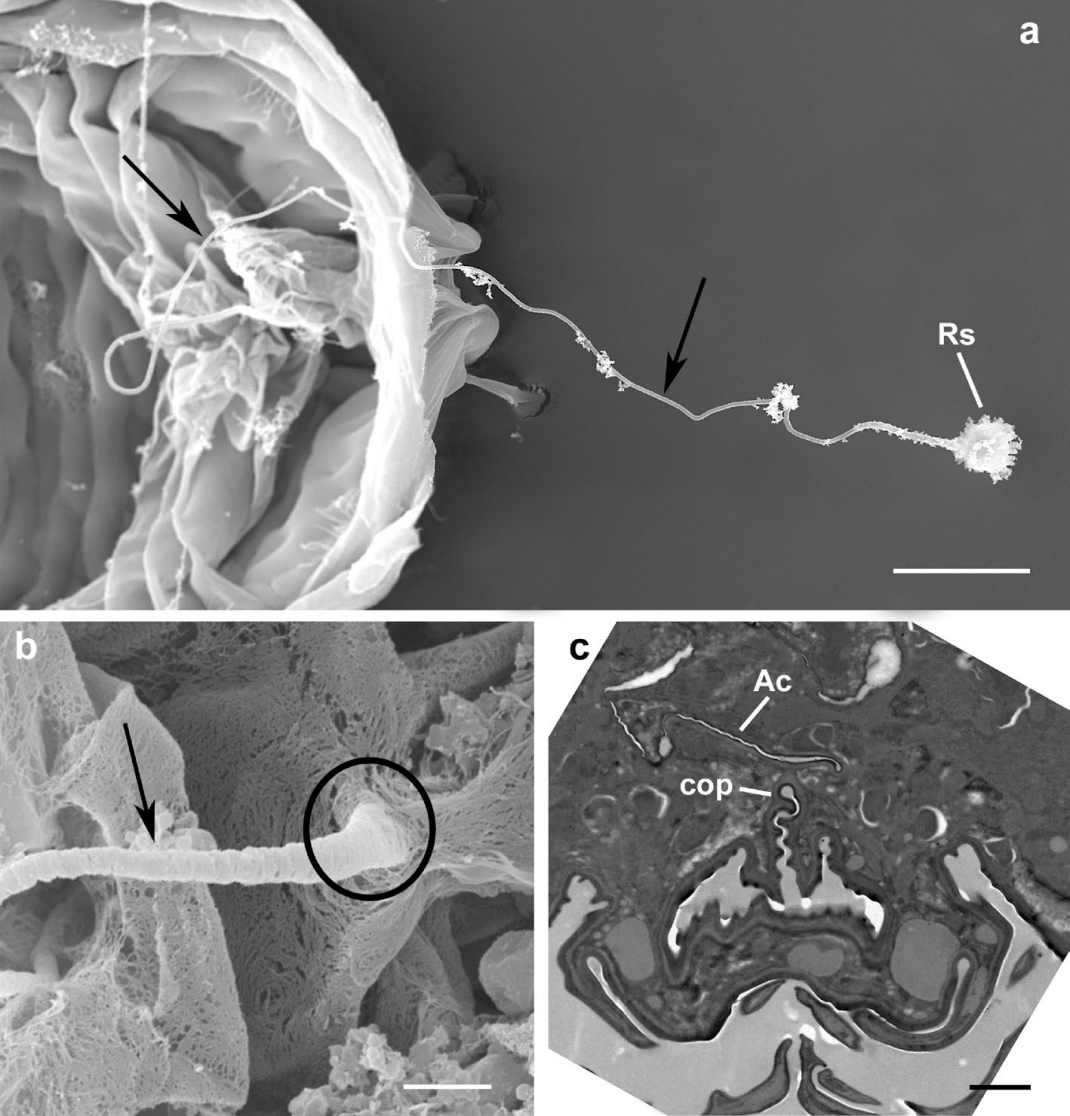
*Brevipalpus papayensis*: insemination system. **a** SEM: overview of insemination system composed of a long insemination duct (arrows) and seminal receptacle (spermatheca, or vesicle); **b** SEM: detail of insemination duct (arrow) close to insemination pore (circle) (view from inside of mite body with dorsal shield removed); **c** TEM: cross section showing copulatory pore ventral to the anal canal. Abbreviations: Ac, anal canal; cop, copulatory pore; Rs, seminal receptacle. Scale bar: **a** 10 µm; **b** 1 µm; **c** 2 µm

The insemination system in *Brevipalpus*, as reported by Pijnacker et al. (1981), Alberti and Coons (1999) and Alberti et al. (2014a), is composed of the insemination duct and the seminal receptacle (spermatheca or vesicle) (Fig. 2a), where the sperm is supposed to be stored.

**Fig. 3:**
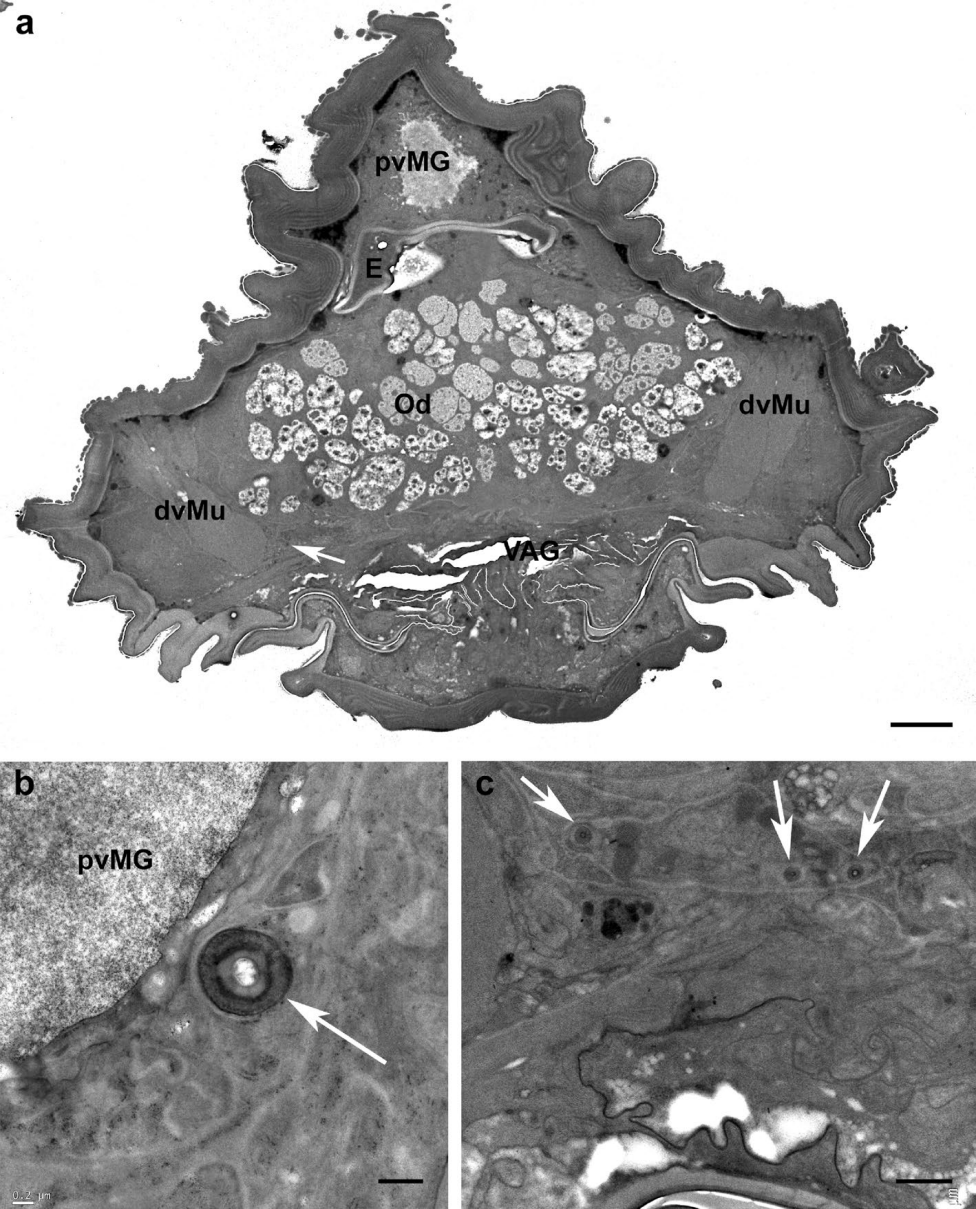
TEM cross sections showing insemination duct (arrow) in different position in *Brevipalpus tuberellus* mite body. **a** Cross section at level of large oviduct showing position of insemination duct (arrow) ventral of oviduct and dorsal of vagina; **b** *B. papayensis*: cross section of insemination duct (arrow) close to postventricular midgut; **c** *B. tuberellus*: cross section showing insemination duct cut several times (arrows). *dvMu* dorsoventral muscles, *E* egg, *Od* oviduct, *pvMG* postventricular midgut, *VAG* vagina. Scale bar: **a** 5 µm; **b**, **c** 0.5 µm

**Fig. 4:**
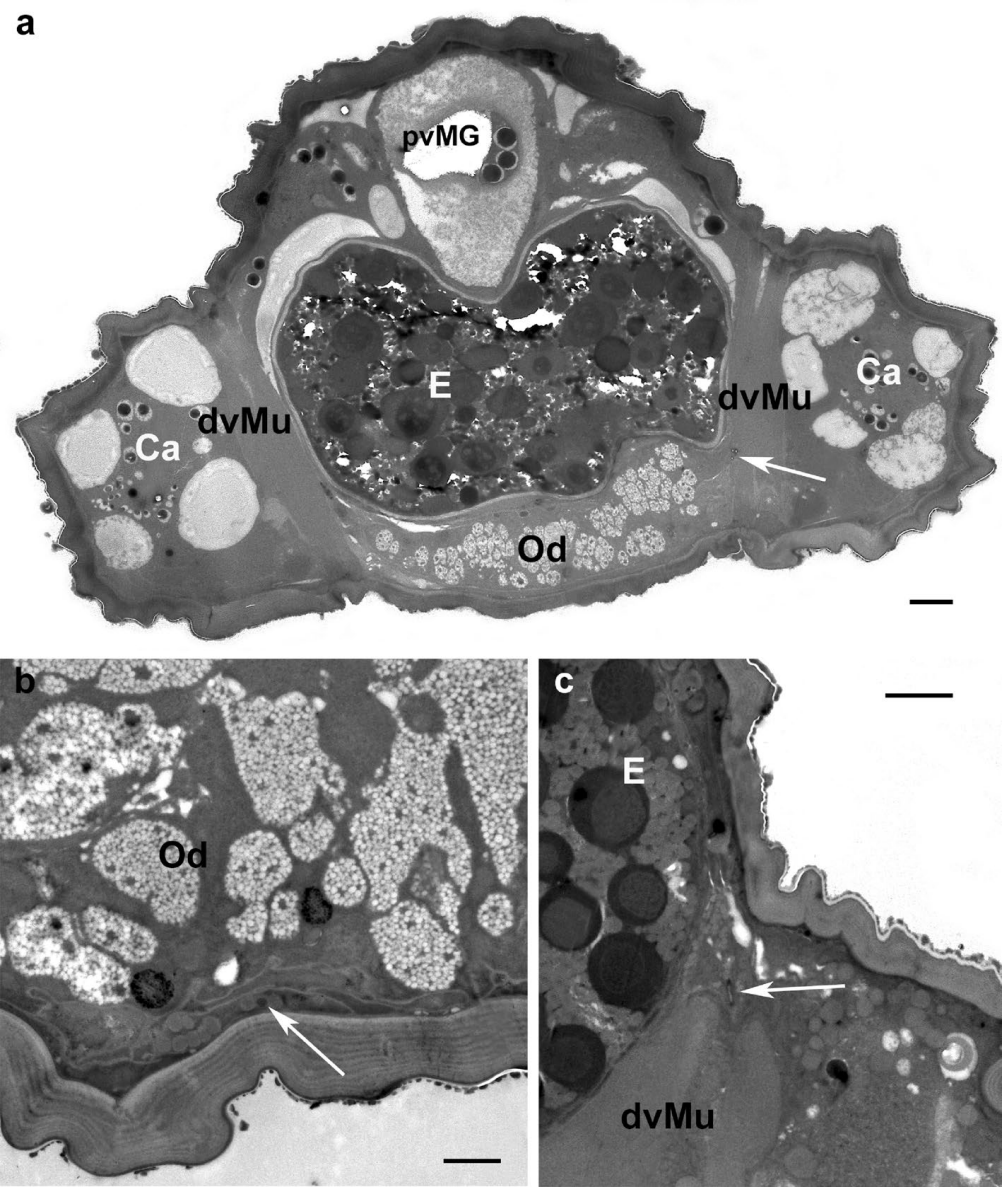
*Brevipalpus tuberellus* TEM cross sections showing insemination duct (arrows) in different positions in mite body: **a** ventrolateral to egg; **b** ventral to oviduct; **c** in dorsal region of body, lateral to egg. *Ca *caecum, *dvMu* dorsoventral muscles, *E* egg, *Od* oviduct, *pvMG* postventricular midgut. Scale bar: **a**, **c** 5 µm; **b** 1 µm

**Fig. 5:**
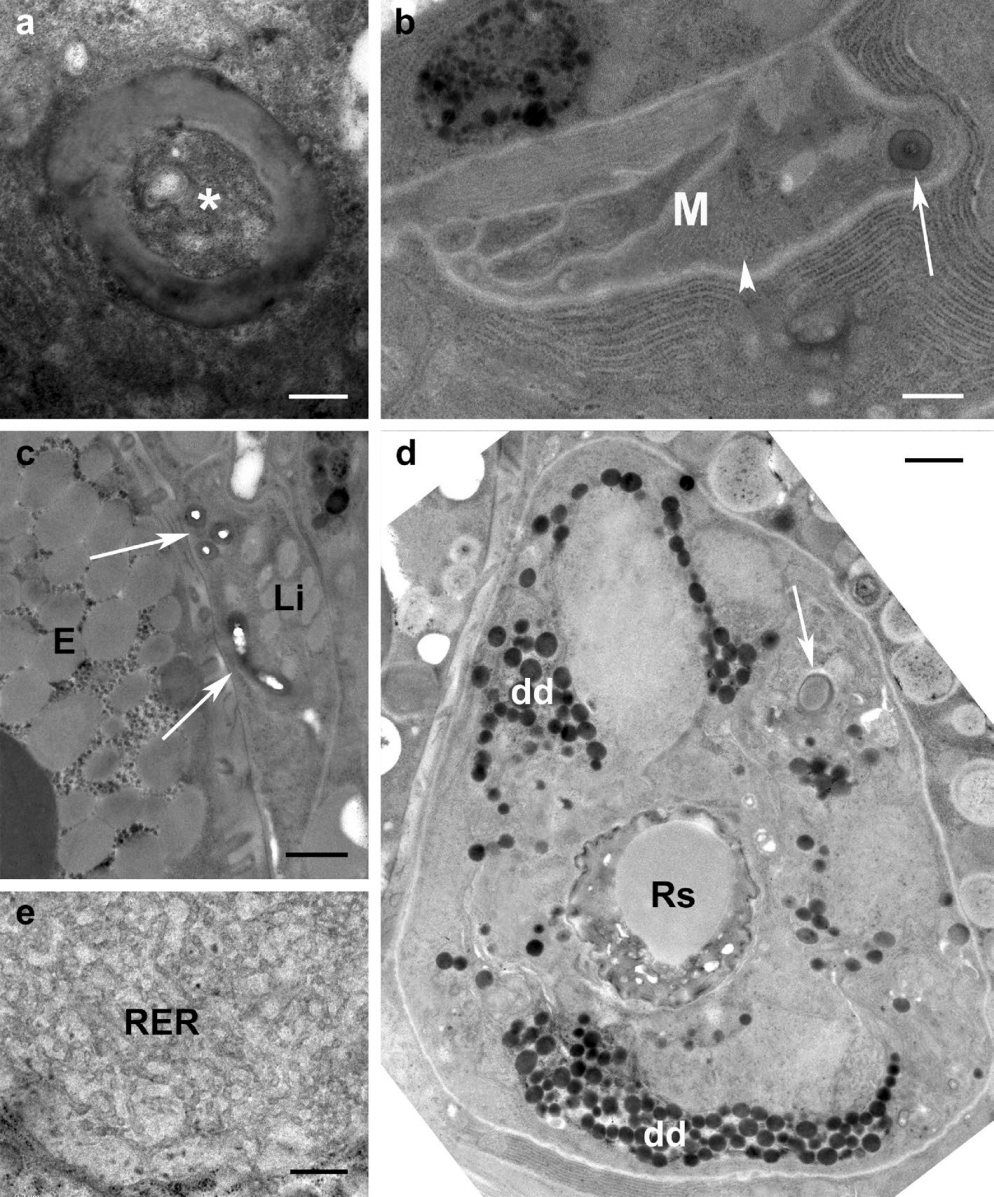
TEM cross sections of insemination duct. **a** *Brevipalpus yothersi*: granular material (asterisk) sometimes visible inside insemination duct lumen; **b**–**e** *B. tuberellus* insemination duct (arrow) embedded in epithelium whose cytoplasm contains microtubules (arrowhead), few mitochondria, ribosomes, lysosomes, dense aggregates of dark droplets, lipid inclusions and enlarged rough endoplasmic reticulum. *dd* dark droplets, *E* egg, *Li* lipid inclusion, *M* mitochondrion, *RER* rough endoplasmic reticulum, *Rs*, eminal receptacle. Scale bar: **a** 200 nm; **b** 0.4 µm, **c**, **d** 1 µm; **e** 0.5 µm

In all species studied with TEM the long insemination duct begins at the copulatory pore (Fig. 2b, c) ventrally located of the anal canal (Fig. 2c) and dorsally of the vagina (Fig. 3a). In cross section, the duct appears round and shows a rather thick cuticle (Fig. 3b) that in the anterior portion of the duct appears bi-layered with an inner (towards the lumen) layer more electron lucent and an external layer (towards the epithelium) more electron dense (Fig. 3b). The insemination duct, so called since it is supposed to be used by the male to introduce the penis (Alberti et al. 2014b) and fertilize the female, runs forward and performs some bending, or undulating, such that it is viewed more than once in some cross sections (Fig. 3c). It is quite difficult to follow, in cross sections, the path of the duct leading to the seminal receptacle due to the very small size of the duct diameter (ca. 0.3 µm; Figs. 3 and 4) and the unpredictable position of the duct in the body. In fact, the duct is pushed aside by the large oviduct, the growing oocytes and the digestive system, so that it can be located dorsally, laterally or ventrally (Fig. 4). Sometimes, a granular material is visible inside the insemination duct lumen (Fig. 5a). As reported by Alberti et al. (2014a), the duct is surrounded by an epithelium that becomes enlarged while approaching the oviduct and the ovary (Fig. 5b–d). The cytoplasm of this tissue enveloping the duct contains microtubules, few mitochondria, ribosomes, lysosomes, dense aggregates of dark droplets, likely of Golgi’s origin, lipid inclusions (Fig. 5b–d), and rough endoplasmic reticulum with enlarged vesicles (Fig. 5e).

**Fig. 6:**
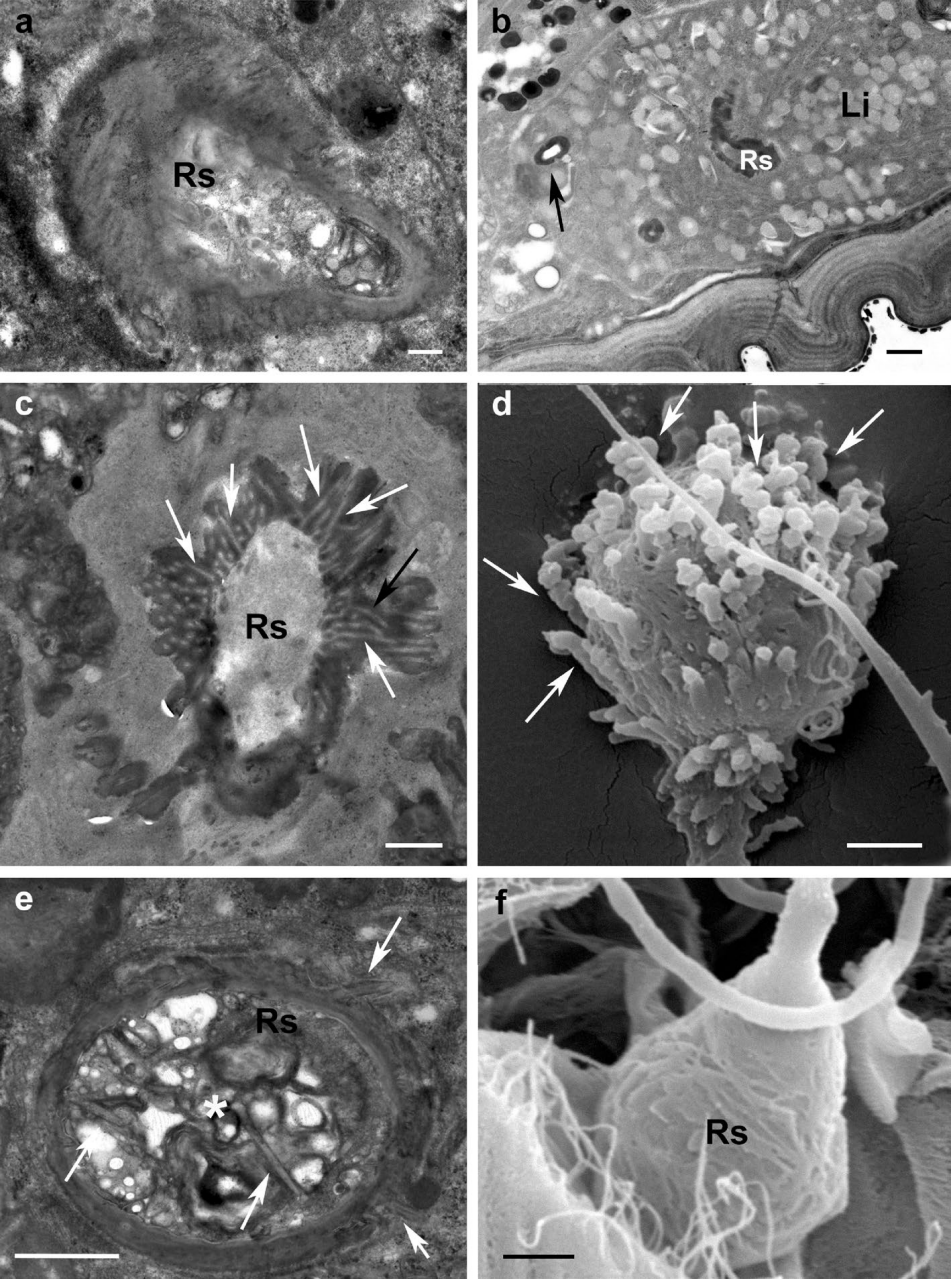
Seminal receptacle. **a** *Brevipalpus yothersi* TEM cross section of insemination duct (arrow) widening to create a seminal receptacle still cuticle-lined; **b** *B. tuberellus* TEM cross section: insemination duct (arrow) and seminal receptacle embedded in thick epithelial tissue showing heterogeneous inclusions; **c** *B. papayensis* TEM cross section: seminal receptacle with a central lumen delimited by a rather thick cuticular layer provided with finger-like structures. Each finger-like structure presents a central thin lumen (arrows) that communicates with lumen of receptacle; **d** SEM, *B. papayensis* seminal receptacle showing evident finger-like projections (arrows); **e** *B. yothersi* TEM cross section of seminal receptacle showing smooth external and internal cuticular layers except for few thin filaments (arrows) projecting into the vesicle lumen and outside the vesicle itself; moreover, some granular and unstructured material is visible inside lumen (asterisk); **f** SEM, *B. tuberellus* seminal receptacle showing external surface slightly irregular. *Li* lipid inclusion, *Rs* seminal receptacle. Scale bar: **a** 0.2 µm; **b**, **c** 0.5 µm; **d**–**f** 1 µm

Close to its end, the insemination duct widens slightly (Figs. 5d and 6a, b) and terminates in the seminal receptacle (also called spermatheca) and still cuticle-lined (Fig. 6a, b). At this level, the epithelium embedding the duct and the spermatheca appears much thicker and achieves a rather complex arrangement of the cells showing heterogeneous inclusions (Figs. 5d and 6b).

In cross sections, the seminal receptacle presents a well visible lumen surrounded by a cuticlar layer that is continuous with the duct cuticle. The thickness and the appearance of the receptacle walls look different in different species. In *B. papayensis* the rather thick and homogeneous cuticular layer forms an irregular external outline projecting in finger-like structures whose lumens connect to the lumen of the receptacle (Fig. 6c). Such finger-like projections are visible in SEM images as well (Fig. 6d). In *B. yothersi* the cuticlar layer is thinner with a smooth external and internal surface except for few thin filaments projecting both outside and into the lumen of the vesicle where some granular and unstructured material is visible (Fig. 6e). In *B. tuberellus* the external outline of the receptacle is slightly irregular whereas the internal surface is smooth (Figs. 5d and 6c). The irregular external surface of the receptacle is visible in SEM images as well (Fig. 6f).

**Fig. 7:**
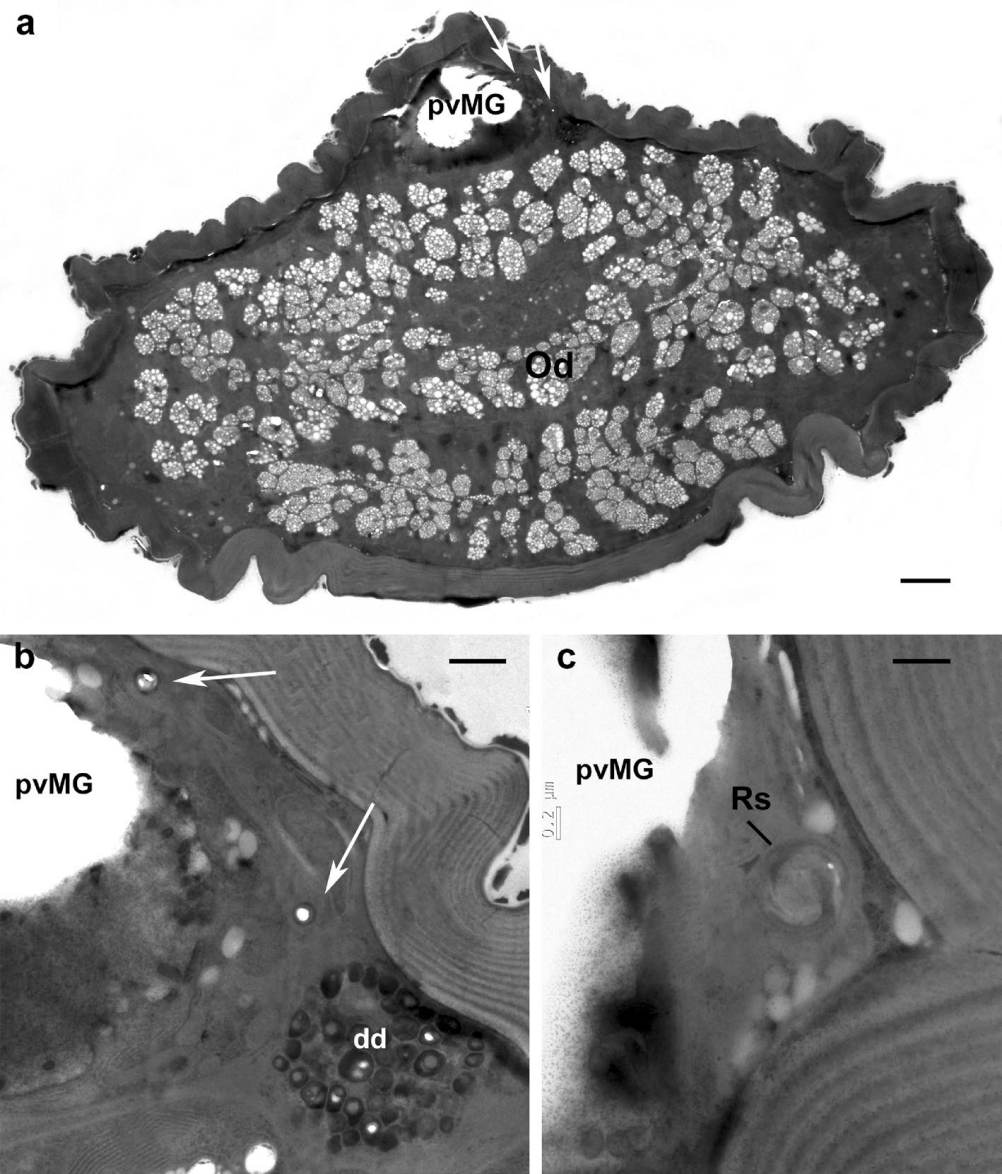
*Brevipalpus papayensis* TEM. **a** Cross section at level of large oviduct showing insemination duct (arrow) cut twice and located dorsal in body on right side of postventricular midgut; **b** detail of previous image, showing duct cut twice and embedded in epithelial tissue with dark droplets; **c** another cross section of same specimen, showing insemination duct ending in a very reduced receptacle (spermatheca) appearing like a small ball of cuticle without any lumen. *dd* dark droplets, *Od* oviduct, *pvMG* postventricular midgut, *Rs* seminal receptacle. Scale bar: **a** 5 µm; **b** 1 µm; **c** 0.5 µm

Remarkably, in some *Brevipalpus* populations, undeveloped seminal receptacles have been observed by LM (A.D. Tassi, unpubl.). Moreover, in the present study a few females of the *B. tuberellus* and *B. papayensis* populations were detected not showing a seminal receptacle when examined under the light microscope. Among the studied females of *B. papayensis*, cross sections of one female (Fig. 7) showed the insemination duct ending in a very reduced receptacle (spermatheca) appearing like a small ball of cuticle without any lumen (Fig. 7c). Nevertheless, the peculiar tissue described as enveloping the insemination duct and the receptacle is still present (Fig. 7a, b).

**Fig. 8:**
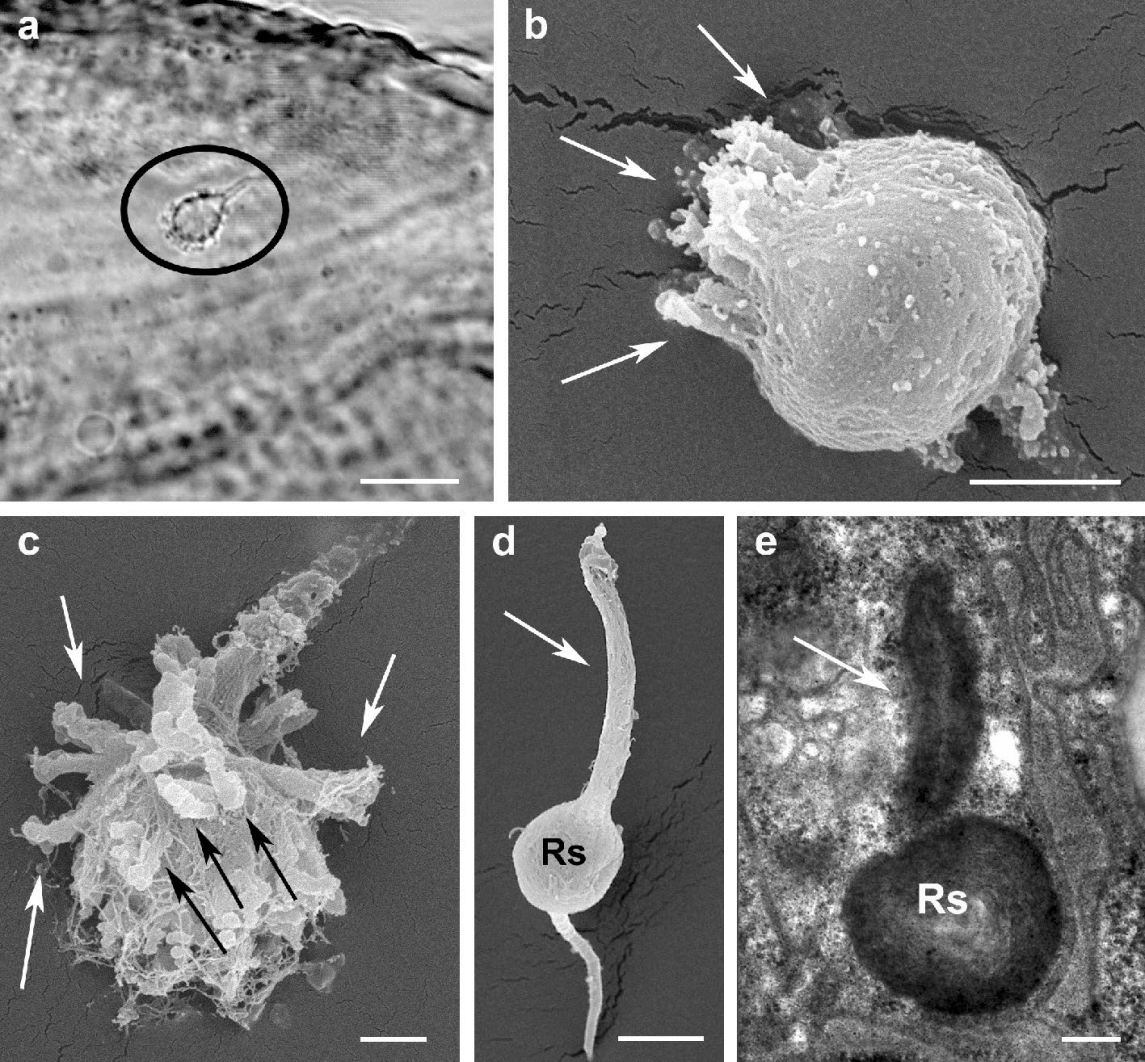
Seminal receptacle. **a** LM *Brevipalpus papayensis* seminal receptacle (circle) with finger-like projections described in TEM and SEM (compare with Fig. 6c, d); **b** *B. californicus* SEM of roundish vesicle with finger-like extensions (arrows) concentrated on distal pole of spermatheca (facing anteriorly); **c** *B. obovatus* SEM of spermatheca similar to *B. papayensis* but with longer, more divergent finger-like projections (arrows); **d**, **e** *B. yothersi* SEM **d** of spermatheca, oval with long tapering apical structure (arrow) visible in TEM section as well (**e**, arrow). *Rs* seminal receptacle. Scale bar: **a** 10 µm; **b**, **d**, **e** 2 µm; **c** 1 µm

**Fig. 9:**
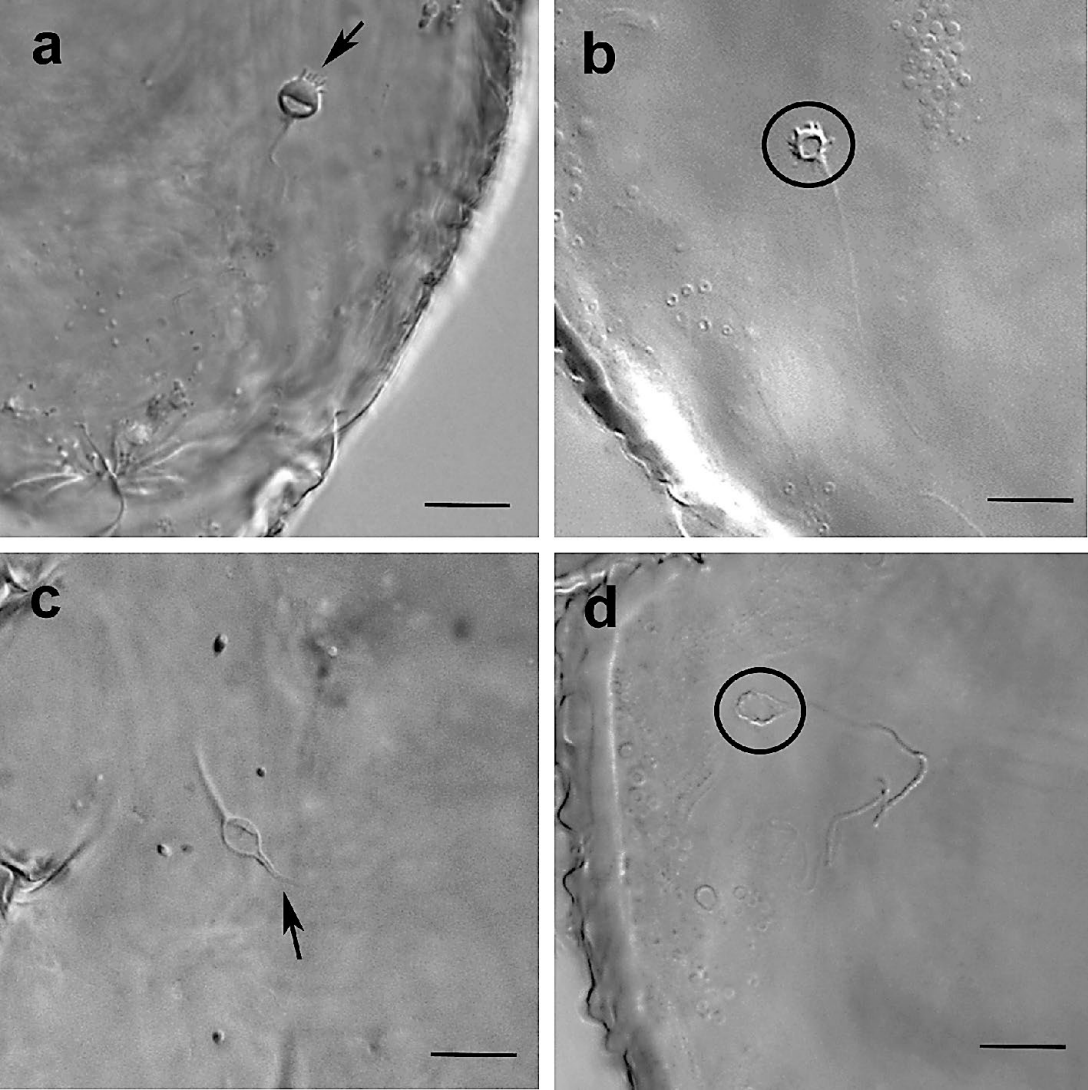
Seminal receptacle (spermatheca) of the studied species in LM. **a** *Brevipalpus californicus*, the vesicle shows finger-like extensions concentrated on one pole (arrow). **b** *B. obovatus*, the spermatheca (circle) presents long, divergent finger-like projections surrounding the external surface. **c** *B. yothersi*, the spermatheca is oval with a smooth surface and a long tapering apical structure (arrow). **d** *B. tuberellus* presents an oval spermatheca (circle) with an irregular external surface devoid of any projections. Scale bar: 10 µm

Recently, Beard et al. (2012) reported the possibility, based on LM observations, that the shape of the seminal receptacles differs between species and even within populations of the same species and within such populations. Thus, beside ultrastructural observations of the insemination system with TEM, SEM investigations were performed to study the external morphology of the seminal receptacle (e.g., size and shape) and to compare these observations with the images obtained by LM. All studied species proved to have species-specific shapes of the spermatheca. In *B. papayensis* the roundish spermatheca is ca. 3–4 µm in size and shows numerous finger-like convoluted projections (Figs. 2a and 6d) that are clearly visible in SEM but discernible in lower magnification with light microscopy as well (Fig. 8a); in *B. californicus*, the vesicle is roundish (ca. 4 µm) but with finger-like extensions concentrated on the distal pole of the spermatheca (the one facing anteriorly) (Figs. 8b and 9a). In *B. obovatus*, the spermatheca is similar to *B. papayensis* (ca. 3.5 µm) and with longer and more divergent finger-like projections surrounding the external surface (Figs. 8c and 9b). In *B. yothersi*, the small spermatheca (ca. 2 µm) looks completely different, as it is oval with a smooth surface and a long tapering apical structure (Figs. 8d, e and 9c). Finally, *B. tuberellus* presents an oval spermatheca (ca. 3.5 µm) with an irregular external surface devoid of any projections (Figs. 6f and 9d).

## Discussion

Five species of *Brevipalpus*, all considered reproduced parthenogenetically, have been studied regarding the ultrastructure of the female insemination system. Remarkably, the ultrastructure of the system proved to be similar to that described by Alberti et al. (2014b) in *B. chilensis* where the males are considered to be functional and thus the population should be reproduced sexually. In fact, even though in the studied species the insemination ducts leading to the receptacles (spermatheca) are very thin, their sizes are comparable to that observed in *B. chilensis* (ca. 0.3 µm); moreover, all receptacles of the studied species are hollow structures with a lumen as in *B. chilensis* (Figs. 5d and 6a, c and e). Thus, it seems that although parthenogenetic, these species have retained the general morphology of the insemination system without significant differences between parthenogenetic and bisexual species. The same phenomenon was observed in mesostigmatic mites of the genus *Veigaia* (Veigaiidae) (Alberti et al. 2009; Di Palma et al. 2012). Veigaiids are peculiar in terms of their reproduction in that males of many species are rarely or never found; yet, the organization of the female reproductive system does not show relevant differences depending on whether being parthenogenetic or bisexual. In fact, in both cases the insemination system and the region involved in egg development and oviposition are similarly organized.

On this basis, and as Ochoa et al. (2011) and Beard et al. (2015) suggested the possibility of using the morphology of the spermatheca as a characteristic to separate species among the genus *Brevipalpus*, the receptacle structure of five species of *Brevipalpus* was investigated by SEM to support the idea that its shape and/or size might be species-specific. In our observations the studied species proved to have a seminal receptacle with a peculiar shape distinctive for each species. As SEM images are three-dimensional and in high resolution, the differences hypothesized by LM studies are confirmed (Figs. 6, 8 and 9). Thus, morphological differences between seminal receptacles of different species are discernable to some extent even with a regularly equipped compound microscope (Figs. 8a and 9). However, a limiting aspect is that for several described *Brevipalpus* species, authors were not able to detect the spermatheca by means of light microscopy, ascribing this to the clearing action of the mounting medium and the young age of the females (Baker and Tuttle 1987). Of course, it is crucial to ascertain whether the seminal receptacle was not detected because of its clarification or reduced sclerotization related to the young age of the females, or because there are some species or populations among the same species that have an undeveloped spermatheca. Cross sections of one female of *B. papayensis* showed an insemination duct ending with a tiny ball of cuticle (ca. 0.6 µm in cross section; Fig. 7c) devoid of any lumen and too small to be detected by LM. This female was collected and processed for TEM together with the others that proved to have a well-developed receptacle. Thus, it seems that among the same species and the same population there might be some specimens whose receptacle may be undeveloped and thus not possible to discern when the mite is slide-mounted.

It is now clear that the inability to detect the spermatheca in some specimens by LM cannot be ascribed to the clarification of the mounting medium or to a reduced sclerotization of this structure related to the young age of the females. Instead, it seems that there are some specimens that have an undeveloped receptacle (Fig. 7c). On the other hand, Alberti et al. (2014a) supposed that differences in LM figures of the shape of the receptacle between populations of the same species and even within such populations may also be due to differences in the state of insemination and structure development. One could hypothesize that differences in physiological condition or age of the females present in the same population or in different populations of the same species can result in various degrees of development of the seminal receptacle (spermatheca) of the insemination system (compare Figs. 6c and 7c, each a cross section of the receptacle of one of two *B. papayensis* specimens). Nevertheless, it is still uncertain how the development of the spermatheca can be modified during the female’s life span. In this respect, in Phytoseiidae (Di Palma and Alberti 2001) and Veigaiidae (Alberti et al. 2009; Di Palma et al. 2012) an epithelium enveloping the vesicle of the sperm access system has been described. Moreover, Di Palma and Alberti (2001) reported that the epithelium appearance varies and that, in some regions, extensive dark inclusions, together with endoplasmic reticulum, free ribosomes, and mitochondria were visible, and that the vesicle wall as well could have different appearances likely depending on the mating state. Also in *Brevipalpus* species, an epithelial tissue is present enveloping the insemination duct and the seminal receptacle (Figs. 5b–e, 6b and 7b). Such epithelium, containing microtubules, mitochondria, ribosomes, lysosomes, dense dark droplets and lipid inclusions, is involved in the secretion of the cuticle wall of the receptacle and thus, likely, in its development and in formation of a normal receptacle or a reduced one. Of course, this hypothesis needs to be tested by LM observations of a high number of females belonging to one species and collected from a single population to support the idea that the development of the spermatheca can be variable, even within the same population, according to different physiological states of the specimens. Moreover, it would be interesting to evaluate the percentage of females showing or not showing well-developed spermathecae within a population and whether such percentages can vary during the seasons or according to some environmental parameters.

Thus, according to the present observations, it seems that the seminal receptacle has specific morphological features that can be useful for taxonomic purposes, yet its appearance within a population might be variable in a way that needs to be ascertained. This is definitely another important step towards successful use of seminal receptacle morphology in the diagnosis of the large genus *Brevipalpus*, as well as of *Brevipalpus* species groups; it may even have diagnostic potential in other genera of the large family Tenuipalpidae.
